# Exploring access to care from the perspective of patients with breast cancer: A qualitative study

**DOI:** 10.1002/cam4.4624

**Published:** 2022-03-10

**Authors:** Carolyn M. Brown, Chisom Kanu, Kristin M. Richards, Laura Stevens, Rahul Sasane, Barbara McAneny

**Affiliations:** ^1^ TxCORE (Texas Center for Health Outcomes Research and Education) The University of Texas at Austin College of Pharmacy Austin Texas USA; ^2^ Research Triangle Institute Research Triangle Park North Carolina USA; ^3^ Innovative Oncology Business Solutions Albuquerque New Mexico USA; ^4^ Cerevel Therapeutics Cambridge Massachusetts USA; ^5^ New Mexico Oncology Hematology Consultants Albuquerque New Mexico USA

**Keywords:** breast cancer, cancer management, psychosocial studies, women's cancer

## Abstract

**Objectives:**

Patients face a myriad of personal and system‐based challenges in accessing breast cancer care, but less is known about access as expressed and experienced by patients themselves. The objective of this qualitative study was to further explore the breadth of issues related to access from the perspective of patients with breast cancer across their care journey.

**Methods:**

Twelve women participated in 1‐h semi‐structured interviews and 48 women participated in 2‐h focus groups at six oncology practices in 2018. Grounded theory was used to analyze the data.

**Results:**

Six primary themes emerged concerning access to care: information, psychosocial support, health insurance, financial resources, timeliness, and emotions.

**Conclusions:**

This study identified six core dimensions of access to care. Access encompassed not only gaining entrée to care services—in the traditional sense of access—but also the continuing support needed to effectively use those services throughout the cancer care journey. Future strategies aimed at improving access to breast cancer care should attend to these ongoing patient‐centric and system‐based issues which are mostly amenable to change.

## Graphical abstract

This study explored access to care from the perspective of patients with breast cancer. Six core dimensions of access to care was identified: support, timeliness, information, health insurance, financial resources, and emotions. Access encompassed not only gaining entrée to care services—in the traditional sense of access—but also the continuing support needed to effectively use those services throughout the cancer care journey.

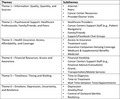

## 1 BACKGROUND

In 2018, an estimated 3,676,262 women were living with breast cancer in the United States, the most common cancer in women aged 20–49 and leading cause of cancer death among women.^1^ Death rates have been declining and 5‐year survival is 90.3%.^1^ However, there are persistent disparities in access to care and outcomes by race/ethnicity and socioeconomic and structural factors.^2^, ^3^, ^4^, ^5^, ^6^, ^7^

Despite efforts to expand health insurance coverage,^3^ many patients experience distress from overwhelming out of pocket expenses, termed “financial toxicity,” after a cancer diagnosis.^8^ They also face many other personal and system‐based challenges, such as social support deficiencies, and logistical issues (eg, wait times).^9^, ^10^, ^11^ Considerable system‐level efforts have also been implemented to improve access to and quality of care in cancer.^12^ One example, the Community Oncology Medical Home (COME HOME) model, showed that improved care coordination reduced utilization and costs, while achieving high patient satisfaction.^13^ Models such as these helped shape the CMS Oncology Care Model (OCM)^12^ and have been important in addressing access from a health systems standpoint, but less is known about access from the patient perspective.

A growing literature on access to breast cancer care as perceived and experienced by patients have confirmed common access challenges such as patient‐provider communication gaps, insurance hassles, financial hardships, uncoordinated care, information deficits, and transportation problems.^14^, ^15^, ^16^, ^17^, ^18^, ^19^ Perceived bias, lack of confidence, and competing obligations are further impediments to care that are prominent among underserved communities.^16^, ^18^, ^20^ Distrust of the healthcare system is common across racial and ethnic minority groups and persists across the breast cancer care continuum.^21^ Family and social support have been reported as key facilitators of accessing and using breast cancer services^22^ and shown to positively impact the prognosis and quality of life of patients with breast cancer.^23^ The purpose of this study was to build upon this burgeoning literature through an exploration of patient's views of access to care across the care continuum from diagnosis through survivorship. The objective of this qualitative study was to further explore the breadth of issues related to access from the perspective of patients with breast cancer across their care journey.

## 2 METHODS

### 2.1 Study design

As part of a larger project that employed a mixed methods research design to develop an access to care scale, semi‐structured interviews (SSIs) and focus groups (FGs) were conducted in 2018. A grounded theory approach using SSIs explored patients' experiences regarding access that would be used to develop the FG moderator guide. The FGs were then used to further explore the broad storylines and identify key concepts that would populate the survey items. Details regarding scale development and testing are available in a separate publication.^24^

### 2.2 Research team and reflexivity

All SSIs and FGs were conducted by a trained interview and FG facilitator who was hired by the principal investigator (CMB). She specializes in market research and has extensive experience in qualitative research. The interviewer/moderator had no prior relationships with the participants and participants were aware that she was external to the research team.

### 2.3 Context

A convenience sample of six oncology clinics served as study sites for patient recruitment. Four of the six sites participated in the COME HOME project and five (excluding California) of the six sites are OCM practices. Clinics were geographically diverse, including locations in Texas (2), Maine, Ohio, New Mexico, and California. All six sites are comprehensive cancer care centers that provide multidisciplinary, team‐based, patient‐focused care across the spectrum of cancers and blood disorders.

### 2.4 Sampling and recruitment

A group of patients across varying demographic and clinical characteristics were recruited by clinic staff at each site via purposive sampling and based on the following inclusion criteria: (1) confirmed diagnosis of breast cancer; (2) 18 years of age or older; and (3) ability to consent. Patients were excluded if they were (1) <18 years of age and (2) did not speak or understand English. All signed a consent form, including permission to audio record for transcription, and received compensation.

### 2.5 Data collection

Twelve 1‐h SSIs were conducted by the trained interviewer/moderator via telephone with patients from four (2‐Texas, 1‐Maine, and 1‐Ohio) study sites, using an interview guide (developed by investigators and reviewed and approved by a practicing oncologist based on her expertise) that consisted of a series of open questions (checklist) with a loose structure to give participants maximum scope for revealing access to care issues (Appendix A). Participants were told the purpose of the study and were encouraged to describe their experiences with accessing care throughout their breast cancer journey. The questions and prompts used in the interviews asked about their experiences with seeking and getting care and about the challenges faced along their care journey.

Semi‐structured interview data were content analyzed by two team members independently via a grounded theory approach^25^ (described below) and five broad themes emerged: (1) experiences in obtaining and receiving diagnosis and treatment information; (2) role of time in diagnosis and treatment; (3) dealing with costs and insurance companies; (4) major areas of support; and (5) feelings and emotions. Results from this thematic analysis were used to develop the FG moderator guide for findings reported in this study.

One 2‐h FG was held at each of the six sites, with 6–10 participants per site. The same interviewer/moderator led all FGs using a moderator guide (developed by investigators and reviewed and approved by a practicing oncologist based on her expertise) that contained open‐ended questions with probes to explore patients' experiences around the five broad areas previously identified in SSIs (Appendix B). Participants were told that “We want to explore the areas of support and challenges you've experienced through your journey with breast cancer. We will tailor our discussion around some broader issues surrounding access to breast cancer care and would like for you to reflect on the particular areas of support and challenges you encountered within these broader issues during your journey.” Both the SSIs and FGs interviews were conducted with unique participants.

### 2.6 Analysis

A grounded theory approach^25^ was used to inductively analyze and identify the themes that emerged from the FGs. NVivo 12 qualitative data analysis software was used to store, organize, and search the coded data. Codes were established using a line‐by‐line open coding technique and a constant comparison method,^25^ and were added or modified as necessary as new meanings emerged.^26^ Axial codes^27^ were formed by relating codes to each other to form themes. Frequencies and means for demographics were obtained using SAS v9.4.

### 2.7 Quality criteria

Two types of trustworthiness were used to ensure data credibility:^28^ (1) negative case analysis to check codes against themes and themes were modified if necessary to include any additional codes; and (2) interpretations of the themes were checked against the raw data to make sure that conclusions were strongly grounded in the data. Also, combining FGs and interviews allowed us to gain a broader range of patient perspectives, and to support the validity of our findings through method triangulation.

### 2.8 Ethics considerations

The larger mixed methods project (2017‐07‐0045) was reviewed and approved on March 2, 2018, by The University of Texas at Austin Institutional Review Board.

## 3 RESULTS

48 women participated in the six FGs. Demographics are summarized (Table 1).

**TABLE 1:** Demographic and clinical characteristics (*N* = 48)

Characteristic	*M* (*SD*)
Age	59.7 ± 11.7

	Frequency (%)^a^
Race/ethnicity	
Caucasian	41 (85.4)
Black	3 (6.3)
Hispanic	3 (6.3)
Native American	1 (2.0)
Geography	
Urban	26 (55.3)
Rural	21 (44.7)
Health insurance type	
Public insurance	27 (57.4)
Private insurance	18 (38.3)
Multiple	2 (4.3)
Stage of breast cancer	
Nonmetastatic	27 (56.3)
Metastatic	21 (43.7)
Diagnosis type	
New diagnosis	29 (60.4)
Recurrence	19 (39.6)
Position in cancer care continuum	
Receiving active treatment	32 (66.7)
Completed treatment	4 (8.3)
Breast cancer survivor	12 (25.0)
Type of treatment received^b^	
Chemotherapy	43 (89.6)
Radiotherapy	24 (50.0)
Surgery	30 (62.5)
Other	11 (22.9)

^a^ Frequencies may not equal *N* = 48 because of missing responses.

^b^ Frequency total is ≥*N* = 48 because of multiple responses.

Six primary themes emerged from the data and primary themes and subthemes are listed (Table 2) and described below, along with exemplary quotes.

**TABLE 2:** Themes and subthemes related to access to breast cancer care

Themes	Subthemes
Theme 1—Information: Quality, quantity, and sources	Internet Peers Cancer center resources Provider/doctor visits
Theme 2—Psychosocial support: Healthcare professionals, family/friends, and peers	Healthcare providers Cancer centers support staff (eg, patient navigators) Family/friends Support/Facebook chat groups
Theme 3—Health insurance: Access, affordability, and coverage	Access to insurance Treatment costs Insurance companies denying coverage Medicare and supplemental benefits Medicaid
Theme 4—Financial resources: Access and awareness	Financial hardship Cancer centers support staff (eg, financial advisor/consultant) Grants Transportation/mobile services
Theme 5—Timeliness: Timing and waiting	Time to diagnosis Time to treatment Wait time between tests/appointments
Theme 6—Emotions: Depression, uncertainty, and resilience	Depression Anxiety/fear Control of outward identity Resiliency

### 3.1 Theme 1—Information: Quality, quantity, and sources

Participants discussed their need for diagnosis and treatment information and sought a wide array of sources to obtain it. The Internet (patient portals, social media, and websites) was a common source. Patient portals were frequently mentioned, but participants also accessed social media and websites to obtain information about breast cancer, look up terms and definitions, and seek information and support from peers with a history of breast cancer. Peers provided clinical information, as well as advice about available resources and services. Oncology practices also served as a key information source. While some patients preferred full information, others found the volume of information to be overwhelming and desired to have better timing of information that coincided with their position in their cancer journey.

I want that information and I think there needs to be stages of it because when I went on breastcancer.org, it has a ton of information, but you have to filter through.

Doctors were an important information source, but the short time spent with providers was a barrier and did not fully allow patients the opportunity to process the information they received.

You don't know until they give you your results and then later, you're like, “Wait a minute, what does that mean?”

You know they don't have a whole lot of time. They've got a lot of patients and so you don't want to hem and haw. You want to just get the two of us to get in and out, whatever. You're home for about 5 h and, “I should have asked this.” That kind of thing. That's happened a lot to me, in this last year … I do wish … that if you had just a brief message you wanted to leave the doctor, not the admin, not the receptionist. They don't have that capability.

### 3.2 Theme 2—Psychosocial support: Healthcare professionals, family/friends, and peers

Participants discussed the importance of support during their cancer journey. The support of healthcare professionals and cancer centers was critical. They cited doctors as a main source of support, which was received positively when compassion, a positive attitude, and even physical contact (eg, “a hug”) were shown. Some participants' satisfaction with care was deeply tied to their access to providers when needed so as not to simply “feel like a number.” Many also emphasized the importance of relationships with nurses and expressed their appreciation for nurses who exhibited sympathy and empathy. One participant verbalized that “nurses are angels with comfortable shoes.” Many identified their cancer center staff as a source of support and resources for other supportive services. The availability and use of patient navigators as resources were instrumental in helping patients “navigate through this” at a time that they are “dealing with a lot.”

Emotional and practical support from family/friends were also valuable to combatting common challenges in cancer care. Patients valued those who accompanied them to appointments, managed day‐to‐day issues, and dealt with procedures.

When I had the mastectomy, my sister came from Oregon and she took care of me and she was the best nurse. That was nice. My family, my daughter lives on the East Coast and she always comes whenever anything happens. She's on the next plane.

[Family support] Very valuable. My friends who would take me to my chemo treatments. You're there for 4 h. It was great to have someone that would either drive you, take you in, bring you back or stay with you, bring a little food, have a little lunch while you were there.

Finally, participants discussed how support groups, including chat groups, provided emotional support, information, and encouragement, and enabled them to connect with others with similar experiences.

For me, support groups. Because even though I have friends/family, support groups. It helps in a way, because family, they don't know what you're going through, so the support of a group, we can identify.

It was fantastic … It was a group of women. We could share our journey. Just like we are sitting in the room now. Any experience you have, you got to hear new things. What their complications were to look out for, and we really supported each other. We had great support … Any questions, any doctor, any whatever. It was all answered out there.

It's good emotional support, information support. There are people that have been there, done that already, or doing it and they understand the frustration, the emotions, all the feelings, the anger, the sadness, the whatever.

### 3.3 Theme 3—Health insurance: Access, affordability, and coverage

Health insurance and the high costs of treatment were major concerns. Many women struggled with accessing and affording health insurance, as well as navigating the health insurance market. Many relied on the advocacy of care coordinators/patient navigators, nurses, or family/friends to handle the complexity of financial issues or petition insurance companies for approval regarding specific medications.

I can tell you, when you feel like you don't have access, those feelings, when you feel like your life depends on it, it's very emotional. It's very upsetting. Like I said, I felt like I was not going to have access. Then my concern coming up, my COBRA runs out at the end of April and I'm going to have to find one of the insurances on the marketplace. I found out there are no PPOs on the marketplace, they're all HMOs, so now I'm going to have to start … First of all, I have to find one that I can afford that … Hopefully I can afford one that I can, number one, keep my doctor.

Treatment costs placed a heavy burden on participants, and even those with “good insurance” were frustrated by high out‐of‐pocket costs. Some applied for and received copay assistance, but copay assistance did not last post‐treatment.

We were able to get the copay relief during treatment. Now treatment's over, I'm getting monthly shots that are $1000 a pop in addition to my oral medication. Insurance is [covering it], as far as the oral med, but that $1000 a month, we don't know who to go to. Because I'm getting that at another facility, that's where we really need help.

Denial of coverage by insurance companies for a variety of reasons (eg, experimental treatment) was also a major source of frustration. Participants described dealing with insurance as a “nightmare.”

A lot of problems, period, getting medication now. No problems with the chemo, but now I'm taking chemo pills, nightmare just to get them.

I did a PET scan, they deemed it not necessary. They said it was experimental.

Some lauded the benefits of having Medicare and supplemental insurance, while others lamented about having to go on public assistance (eg, Medicaid). Those denied public assistance were left to struggle with unpaid medical bills and a sense of hopelessness.

Well you're only a family of four at $50,000, you're right there in the poverty line. You don't qualify for Medicaid anymore and … Then because I'm on oxygen at night, I've got my oxygen bills I'm paying for and I've got these bills. They call me, collections, and I say, “Well I'm a cancer patient, if you'd like to come collect my body you can.” What else am I going to tell them?

### 3.4 Theme 4—Financial resources: Access and awareness

The high cost of treatment caused financial toxicity for many. They described depleting their savings and retirement funds, selling their homes or belongings, struggling to pay bills, and losing their jobs along with the associated worry and stress “on top of trying to get well.” Utilizing financial advisors at their clinics were helpful for obtaining grants for medical expenses. However, participants were frustrated by the fact that they were often unaware of this resource until well into their treatment and stressed the need for financial support at the very beginning of, during, and after treatment.

I did not have an experience with a navigator. I have talked with a financial consultant, but that was far into my experience. I would have liked to have talked to her a lot earlier (laughter) … I commented about how expensive the medicine was that I was getting. My doctor just took that. I didn't realize there even was a financial consultant … They should know that it's expensive for everybody, whether or not you make a lot of money or you don't, it's expensive. It doesn't matter if you make $0 or you make $250,000, it's expensive and they should afford those options … Everybody should talk to someone.

Transportation for treatment was also an issue described by participants. Some were not always comfortable asking friends and family for assistance but noted that getting rides from free medical transportation services was helpful.

### 3.5 Theme 5—Timeliness: Timing and waiting

Timing was a critical issue, and it intersected with many other themes. Participants discussed the amount of time between the initial doctor's appointment, diagnosis, referral, and treatment initiation. Some highlighted the emotional difficulty of waiting to receive test results and start treatment following diagnosis. Medical referrals and long wait times, especially in specialist or doctor shortage areas, were very difficult given their sense of urgency and uncertainty about the next steps in their diagnosis and treatment.

I don't know about anybody else, but for me, one of the hardest, if not the hardest was the waiting to find out my diagnosis.

I wanted to have it done the next day. I was a little bit shocked, too, when they came back and said, “He can see you at the end of January,” and I thought, that's a whole month. In my mind this is pretty serious stuff and urgent. I don't want to wait a month.

I have a gut problem and so it's an issue of chemo. I need to start chemo right now, but I can't see GI for three months.

### 3.6 Theme 6—Emotions: Depression, uncertainty, and resilience

Participants experienced a range of emotions throughout their cancer journey. They discussed feelings of depression, anxiety, fear, loss of outward identity, survivor identity, spiritual beliefs, and resiliency. Intense emotions tended to occur episodically throughout their journey, while some negative emotions diminished as treatment progressed and familiarity with the process increased. Anxiety and depression coincided with the uncertainties surrounding cancer and were expressed by patients regarding the lag time between testing and obtaining results, the nature and severity of chemotherapy side effects, and the management of their treatment among other responsibilities. They discussed the role of family/friends in helping them through depression, as well as depression experienced by family members because of the patient's cancer diagnosis—an often‐overlooked aspect of cancer care.

I'll tell you about depression, it has affected my husband a lot more than it has me … We deal with it, we process it, we process those emotions, fears, worries, concerns and then we recover, and we go on. My husband recently had to get on some antidepressants to help because someone helped him realize, “You're grieving your wife's death and she's not even dead.” He's been on it now for a month and it has made a world of difference. I wish we'd have done it so much sooner. We fail to recognize a lot of times, I think, the family members' depression and what they're going through.

While participants experienced a loss of control of their outward identity while undergoing treatment and felt a loss of who they are, they were proud of their resiliency and strength despite their cancer and treatments.

I think it's so hard when you have the treatment and you lose your hair, eyebrows, eyelashes. Not only do you have cancer, but now the world knows it. I just want to be [name], but you're [name] with cancer to the world.

The first thing people look at me and say she has cancer. I want to still just be [name].

I've tried many different chemotherapies. They work for a while and then they stop. They keep putting me on different things. They work and then they don't. Now I'm on one that's kicking my butt, but I won't let it (laughter). I go to work the day after. I work out constantly. I keep myself healthy, so this thing won't destroy my life.

## 4 DISCUSSION

Access to care is a multidimensional concept and this study identified six core dimensions of access from the perspective of patients with breast cancer. There were two overarching themes of support and timeliness which intersected the other four themes of information, health insurance, financial resources, and emotions. While emotions are not traditionally seen as a component of access, our findings demonstrate that meeting the psychological and emotional needs of patients with breast cancer is directly related to the “goodness of fit” of care for patients which will determine their acceptability and use of services.^29^

The importance of information among patients was paramount, which is consistent with previous findings of high health information needs in patients with breast cancer.^19^, ^30^ Participants received and sought diagnosis and treatment information from a variety of sources, including healthcare providers, peers, and the internet. While patients rely on providers for information personal to their own care, they are also proactive information seekers and self‐advocates thanks to the increased accessibility of information and peer groups on the internet. However, some were overwhelmed and preferred timing information provision relative to their position in their care journey (a sort of “stage‐matched” information process), which may in turn facilitate their understanding next steps, setting expectations, and reducing uncertainty associated with treatment. Providers could be more intentional about providing information tailored to “meet patients where they are” on the care continuum.

Patients' psychosocial and practical support needs were also prominent themes. Extant research shows that patients with cancer often have unmet psychosocial needs.^19^, ^31^ Moreover, issues such as financial hardship, insurance, and transportation (referred to as “little big things” by patients with ovarian cancer)^10^ represented critical access to care concerns for patients in this study as well. Providers, support staff, family/friends, and peers were all important sources of social and instrumental support in navigating the healthcare system and mitigating barriers. Patients acknowledged that the limited time spent with providers was sometimes problematic, but they also relied heavily on clinic staff for support and referral to services. However, patients were not always made aware of resources (eg, financial services) available to them in a timely manner, if at all. Patient navigators, shown to be valuable to cancer care outcomes,^11^, ^32^, ^33^, ^34^ were endorsed by patients in our study and represent a requirement of OCM practices to ensure patients receive the services they need. Roche et al.^10^ offered a sample care team map, as an initial step, to delineate resources within a system as one way to alert patients to resources available to them. Our findings indicate that meeting these resource needs are paramount to patients successfully navigating the cancer care continuum.

Family/friends provided social, emotional, and practical support as well as advocacy in helping patients cope with cancer and treatment, and likewise assisting with transportation, daily activities, and insurance hassles. However, patients were cognizant of not becoming a burden. Peer groups offered useful information and social support, shared experiences with navigating life with breast cancer and provided a means of networking. Peer social support has been shown to decrease depression, promote psychosocial well‐being and sense of community, and foster exchange of information/experiences.^34^, ^35^, ^36^, ^37^

Finally, our findings indicated that timeliness of information and care was also a prominent theme of access. Waiting for treatment initiation, test results, and referrals were especially trying experiences, and could be even more pronounced in ethnic/racial minorities with limited support and/or resources.^38^ Even in a program designed to reduce access barriers, timeliness of care persisted for non‐Hispanic black and Hispanic patients.^39^ Patients discussed the roles that family/friends and providers played in helping them deal with negative emotions that usually accompanied the diagnosis/treatment of breast cancer. Negative emotions of family members, an often‐overlooked aspect of cancer care, was also discussed. Spiritual beliefs and resilience helped some better cope with their breast cancer. Dealing with negative emotions is not uncommon in patients with breast cancer and their caregivers.^35^, ^36^, ^37^, ^40^, ^41^, ^42^ Yet, caregivers and family/friends do not receive sufficient advice and training to support patients and take care of their own health.^43^ Being mindful of the psychological and emotional state of patients as well as their caregivers can enable providers to better meet patients' needs by customizing patient‐provider interactions and/or referring patients to mental health services, as appropriate.^44^

### 4.1 Study limitations

Potential limitations of this study involve generalizability. The purpose of qualitative research is often to explore the landscape rather than to measure impact. Accordingly, it is possible that issues identified may not represent the universe of potential access to care issues experienced among patients with breast cancer. However, we attempted to mitigate this limitation through the recruitment of a diverse, heterogenous sample across six geographically diverse oncology practices. Our sample was mostly Caucasian, so findings likely do not fully reflect access from the perspectives of ethnic/racial minorities. In fact, issues of culture, bias, and racism were not found in our study but have been in previous studies of access in cancer.^16^, ^18^, ^20^ Their absence could be a positive reflection of our study sites. Another limitation is that our sample included only women, so findings may not represent issues important to men with breast cancer. In addition, the oncology practices in our study are considered high functioning sites regarding patient care, with all but one being an OCM practice. Therefore, access issues relevant to patients of other types of oncology practices with fewer resources may not be represented. Finally, our research team consisted of academics and practitioners with deep expertise in research methods including qualitative research and oncology. Our extensive experiences in these areas could have impacted our interpretations of the data, although we attempted to minimize bias by having a trained independent consultant facilitate data collection.

### 4.2 Clinical implications

While the importance of support and timeliness in access to care is not a novel finding, our results show that support and timeliness are intersecting factors across other dimensions of access to care—information, health insurance, financial resources, emotions—as experienced by patients with breast cancer. First, practices should implement multidisciplinary, support programs, including navigation services, that address all components of access to care, with attention to the tailored, timely, and ongoing provision of information and services that support patients throughout the care journey. Services provision and care need to be nimble and responsive to patients' challenges during all stages of cancer care. Patients desire information but may only want information particular to their needs at a certain time. Ideally, caregivers could be a part of educational efforts to help them learn how to better support patients while taking care of their own health. In addition, all patients should be made aware of every available service to them from the start. Full‐scale support early on could be even more crucial in disadvantaged populations that may have insufficient support or resources to fully benefit from care. Finally, we recommend that efforts to reduce time to diagnosis and wait times for services be a priority. Given the often devastating mental health impact of a cancer diagnosis, timely access to care and services could help to mitigate patients' emotional challenges and improve their resilience from diagnosis through survivorship. The success of COME HOME and other OCM prototypes in addressing many of these access issues can provide a blueprint for other practices to adopt similar processes.^12^, ^13^

## 5 CONCLUSIONS

This study identified six core dimensions of access to care from the perspective of patients with breast cancer—two primary components of support and timeliness which intersected the other four components of information, health insurance, financial resources, and emotions. Access encompassed not only gaining entrée to care services—in the traditional sense of access—but also the continuing support needed to effectively use those services throughout the care journey. Future initiatives aimed at improving patients' access to breast cancer care, understanding their issues, and addressing their challenges should attend to these ongoing patient‐centric and system‐based issues which are mostly amenable to change.

## CONFLICT OF INTEREST

The authors have no potential conflicts of interest to report.

## AUTHOR CONTRIBUTIONS

Carolyn M. Brown, Kristin M. Richards, Chisom Kanu, Laura Stevens, Rahul Sasane, and Barbara McAneny contributed to the conception and design of study, data acquisition, interpretation of results, and manuscript review and edit. Carolyn M. Brown contributed to data analysis and was responsible for writing the draft manuscript. Carolyn M. Brown and Rahul Sasane were responsible for funding acquisition. Kristin M. Richards and Chisom Kanu contributed to project management. All authors read and approved the final manuscript. All authors agreed to be accountable for all aspects of the work.

## ETHICS STATEMENT

The University of Texas at Austin Institutional Review Board approved this study (Approval number: 2017‐07‐0045).

## Data Availability

The data that support the findings of this study are available from the corresponding author upon reasonable request.

## APPENDIX A

### SEMISTRUCTURED INTERVIEW GUIDE

#### Diagnosis of breast cancer

[See brackets for Reoccurrence of breast cancer diagnosis]

- When you were first diagnosed breast cancer [or When your breast cancer reoccurred], what was your experience with getting care? Let us continue first by extending on what you shared in the introduction surrounding thoughts and feelings.

*Potential probes*:

Feelings:

- Anxiety or fear regarding diagnosis [or reoccurrence] and death
- Anxiety or fear regarding treatment/side effects

Thoughts:

- Consideration/interest in or sought other forms of care—such as complementary and integrative therapies
- Patient beliefs—such as beliefs about fatalism regarding cancer [or reoccurrence]
- Social support

- More specifically, what were the challenges you faced when seeking care?

*Potential probes*:

- Socio‐psychological issues
  - Social support
  - Cultural incompatibility
  - Life stressors
  - Language barriers
  - Religious/spiritual beliefs
- Geographic—rural/urban, distance, transportation
- Affordability—insurance, co‐pays
- Clinical issues
  - Type of treatment required
  - Availability of treatment
  - Stage at diagnosis
  - Time of diagnosis (winter vs. summer)
  - Other comorbidities
- System issues
  - Referral system (public vs. private)
  - Hospital/clinic characteristics—type of hospital, hospital size
  - Availability of cancer specialists
  - Type of insurance accepted by cancer specialist
  - Inconvenient clinic hours
  - Quality of care transition
  - Availability of patient assistance programs
  - Availability of support groups
  - Availability of ancillary services (ie, nutrition, social worker, navigator, etc)
  - Provider or clinic delay in follow‐up
  - Quality of pathology reports
  - Quality of radiology reports
  - Insurance delays (approving scans, authorizing treatment, required referrals, etc)

- How did that change, if at all, as you received and throughout treatment?
- What would you say was the biggest challenge in terms of getting information? Why is that would you say?
- What would you say was the biggest challenge in terms of access to resources? Why?
- What would you say was the biggest challenge in terms of seeking care? Why?
- What are your opinions/feelings about the care you received?

*Potential probes*:

- Quality of interaction with healthcare providers
  - Perceived disrespect/Racism
  - Satisfaction with care
- Any issue with (1) referral system (public vs. private) and/or (2) hospital/clinic characteristics—type of hospital, hospital size

- Today and now, from the “inside looking out” would you say that you, as a breast cancer patient, have access to the information, resources, and care that you need? If yes, why? If no, why not?

## APPENDIX B

### FOCUS GROUP MODERATOR GUIDE

We want to explore the areas of support and challenges you have experienced through your journey with breast cancer. We will tailor our discussion around some broader issues surrounding access to breast cancer care and would like for you to reflect on the particular areas of support and challenges you encountered within these broader issues during your journey.

1. First, let us talk about your experiences in receiving and obtaining **information** along the way.
  - Where did you get information?
    - Probe: patient portals, online, family, healthcare providers
  - Was the information helpful? Why or why not? What could have made it more useful?
    - Probe: relevant to person “for me” or stage of treatment, timing of information, expectations, reduce uncertainty
  - What got in the way of obtaining important information?
    - Probe: relevant to person “for me” or stage of treatment, timing of information, amount of information, uncertainty
  - Did you feel knowledgeable after obtaining information? Why or why not?
    - Probe: transition of information to knowledge → care confidence relevant to person or stage of treatment, timing of information, expectations
2. Next, let us talk about the role of **time** in your experiences of diagnosis and treatment.
  - What was the timing like between various diagnostic services and starting treatment?
    - In what ways was it timely or delayed?
      1. Probe: waiting for appointments, referrals, scheduling, care coordination
    - What did you know about the next steps?
      1. Probe: uncertainty, consistency, care coordination, communication among providers
  - What did you think about the travel time it took to get to your various doctors (eg, to drive to the various locations)
    1. Probe: proximity, family/practical support, consistency, transportation
  - How did access to online information make things more or less efficient for you?
    1. Probe: online patient portals, cancer care information, support groups
  - What parts of your journey felt slow and why?
  - What parts of your journey felt fast and why?
3. What kinds of things made it harder or easier to deal with **costs and insurance** companies during your journey?
  - Who/what helped you?
    - Care coordinator, pharma support line, online sources, family/friends, healthcare providers
  - How did these things help?
  - What would have happened without them?
  - Who/what made it hard?
  - How did you handle this challenge?
4. Now, we are going to discuss major areas of **support** you encountered along the way. These can be family and friends as well as healthcare workers. Tell me about your major areas of support / specific areas of support during your journey.
  - How did this support you?
    - Probe: social support, practical support, emotional support, clinical support, financial support, care coordinator, doctor/s (oncologist, radiologists, etc.)
  - What would have been different without this support?
  - Given your look back on this support, is there anything you would do differently (eg, access it earlier or more often?)
  - What kind of support was missing?
    - How would that support have helped you?
5. Let us talk a few minutes about your **feelings and emotions** through your journey. How would you describe your feelings and emotions along the way?
  - How have they affected your access to care along the way, if at all?
    - Probe: Uncertainty about outcomes or side effects, anxiety, stress, burden.
